# Construction and validation of a phenotypic prediction model for bacterial gentamicin resistance using deep learning with gene sequences

**DOI:** 10.1128/spectrum.01906-25

**Published:** 2026-07-15

**Authors:** Jun Li, Siyan Xue, Lingxuan Hou, Zhixuan Zhang, Kaixuan Yuan, Xiaozhong Chen, Chui Kong, Liyang Wang, Bing Gu, Xiaoxiao Liu

**Affiliations:** 1 Laboratory Medicine, Guangdong Provincial People’s Hospital (Guangdong Academy of Medical Sciences), Southern Medical University 89346, Guangzhou, China; 2 School of Biomedical Engineering, Tsinghua University 12442, Beijing, China; 3 Optical Bioimaging Laboratory, Department of Biomedical Engineering, National University of Singapore 193220 , Singapore, Singapore; 4 School of Medicine, South China University of Technology 506909 , Guangzhou, Guangdong, China; 5 School of Information Science and Technology, Fudan University 540392 , Shanghai, China; Shenzhen University School of Medicine, Shenzhen, China

**Keywords:** prediction model, antibiotic resistance, deep learning, genomic, *Klebsiella pneumoniae*

## Abstract

The emergence of bacterial resistance to antibiotics poses a significant threat to human health; thus, there is an urgent need for new strategies in understanding the mechanisms of resistance and further fast prediction of it. Deep learning models offer promising solutions through analyzing genetic sequences in the prediction of bacterial resistance patterns. This study develops and validates a transformer-based deep learning model, DNABERT-2-117M, to predict gentamicin resistance in *Klebsiella pneumoniae* directly from whole-genome sequences. Our central methodological advance investigates the impact of the DNA tokenization strategy on predictive performance. We prospectively compared a dynamic tokenization approach against conventional fixed-length tokenization. Evaluated through rigorous fivefold cross-validation and on a hold-out test set, the model employing dynamic tokenization achieved superior performance, with a mean F1-score of 0.95 and an area under the curve of 0.97. Our findings establish that optimizing sub-sequence tokenization is crucial for model accuracy, and this dynamic tokenization approach significantly enhances model accuracy for antibiotic resistance prediction from genomic data. This genome-based predictive model represents a scalable and rapid alternative to traditional antibiotic susceptibility testing, offering the potential to accelerate clinical decision-making and improve patient outcomes in managing *K. pneumoniae* infections.

**IMPORTANCE:**

This study addresses a critical gap in diagnostic technologies for hypervirulent, antibiotic-resistant *Klebsiella pneumoniae*. We introduce a transformer-based deep learning framework that utilizes dynamic tokenization strategies to predict drug resistance directly from genomic sequences. The core significance of our work is the development of a robust genomic prediction model that serves as a foundational component for future diagnostic paradigms. The significance of this work lies in its potential to fundamentally alter clinical timelines. By decoupling resistance prediction from the requirement for phenotypic growth, our approach is a critical step toward next-generation workflows (e.g., clinical metagenomics) that could deliver a complete diagnostic and susceptibility report directly from a patient sample within hours. This represents a scalable, rapid diagnostic platform that promises to accelerate the administration of targeted treatment for high-risk *K. pneumoniae* infections, with a clear trajectory toward same-day, specimen-to-result diagnostics in the near future.

## INTRODUCTION

Antimicrobial resistance (AMR) has emerged as a grave public health issue endangering human health, with resistant infections directly causing an estimated 1.27 million deaths annually worldwide (1). A key driver of this crisis is the dissemination of antibiotic resistance genes (ARGs) (2, 3) among pathogens via mobile genetic elements, a process accelerated by antibiotic overuse (4). Among the most concerning pathogens is *Klebsiella pneumoniae*, a major member of the *Enterobacteriaceae* family, responsible for severe infection of both nosocomial and community-acquired. *K. pneumoniae* is an opportunistic Gram-negative pathogen that represents a significant global threat due to antimicrobial resistance. According to the 2019 global burden analysis, *K. pneumoniae* was the second most common cause of AMR-attributable deaths, following *Escherichia coli*, underscoring its critical clinical importance (5, 6). The emergence of hypervirulent *K. pneumoniae* (HvKP) strains poses a severe challenge to clinical treatment and patient safety (7). *K. pneumoniae* has been divided into two (8) groups based on their pathogenicity: HvKP, characterized by its ability to cause severe, community-acquired invasive infections even in healthy hosts (9, 10), and classical *K. pneumoniae* (cKP), which typically causes opportunistic, hospital-acquired infections in immunocompromised individuals and is often multidrug-resistant (11). Historically, cKp is prone to be with low pathogenicity but high antibiotic resistance, whereas HvKp is the opposite (12). However, genetic exchanges between cKp and HvKp have resulted in HvKp isolates resistant to most or all available antibiotics, such as carbapenem antibiotics, which pose a major challenge to public health (13). Hence, a rapid and accurate method to identify *K. pneumoniae* and detect its drug-resistant isolates is urgently needed.

Whole-genome sequencing (WGS) coupled with machine learning offers a promising alternative to culture-based antibiotic susceptibility testing (AST). While machine learning models have been successfully applied to predict AMR in other pathogens like *Mycobacterium tuberculosis* and to discover novel antimicrobial peptides (14), their application to predict phenotypic resistance in *K. pneumoniae* from genomic data is still in its nascent stages. Transformer-based deep learning models, in particular, are well-suited for genomic analysis as they can effectively capture the complex, long-range dependencies within DNA sequences that may underlie resistance mechanisms, an advantage over conventional machine learning approaches. For example, it was reported that by using a machine learning approach, a total of 63,410 microbial genomic data sets and 87,920 high-quality microbial genomes from the ProGenomes2 database were input, resulting in the output of a new database, Sphere, targeting nearly 100,000 antimicrobial peptides (15). Justine Riti et al. combined deep learning with microfluidics to characterize bacterial growth and drug sensitivities. Their DropDeepL AST method (a droplet- and deep learning-based AST approach) determines susceptibility profiles for 21 fast-growing *E. coli* and *K. pneumoniae* isolates, providing clinically relevant drug sensitivity results in under 2 h (16). Similarly, Mohammadali Serajian developed a machine learning classifier for rapid identification of drug resistance phenotypes in *M. tuberculosis* using both linear and nonlinear models (17).

Although machine learning has been widely applied to analyze tumor markers and bacterial drug resistance, studies specifically focused on predicting drug resistance phenotypes in *K. pneumoniae* remain limited. Additionally, the impact of model architecture choices, such as the DNA tokenization strategy, on performance is underexplored. Therefore, the primary objective of this study was to develop and validate a transformer-based deep learning model for predicting gentamicin resistance in *K. pneumoniae* directly from WGS data. A key goal was to systematically evaluate how different tokenization strategies—specifically, dynamic versus fixed-length tokenization—affect the model’s predictive accuracy, robustness, and computational efficiency. Tokenizer is the algorithm that segments a raw input sequence (such as text or DNA bases) into smaller, discrete units called tokens. These tokens are then used as the basic input elements for a model. In our study, tokenization allows the model to handle large genomic data flexibly while preserving biologically relevant context.

In this study, we transition from conventional machine learning to a more powerful, pre-trained transformer-based deep learning architecture. Transformer models are uniquely suited for genomic data as they can capture complex, long-range dependencies within DNA sequences that may govern resistance phenotypes. This study introduces and validates a novel model for predicting gentamicin resistance in *K. pneumoniae* directly from whole-genome sequences. Critically, we move beyond simple model application and conduct a systematic investigation into the impact of DNA tokenization—a fundamental step in genomic data processing—by comparing dynamic versus fixed-length strategies. Our work aims to establish a robust predictive framework and provide crucial insights into optimal model design for genomic AMR prediction.

## MATERIALS AND METHODS

### Data collection

From February 2018 to May 2019, a total of 98 consecutive, non-duplicate *K. pneumoniae* clinical isolates were collected from 86 pediatric patients in the pediatric ward of the Affiliated Hospital of Xuzhou Medical University, China. Most patients were diagnosed with pneumonia or sepsis. The bacterial strains used in this study are detailed in Table S1. Importantly, this cohort reflects the high endemicity of carbapenem-resistant *K. pneumoniae* within this specific clinical unit during the study period and was not selected to represent the overall institutional resistance prevalence. Among the 98 isolates, 72.4% originated from sputum, 9.2% from blood, 8.2% from urine, and 3.1% from ascites. These isolates were classified into 15 sequence types, with ST11 being predominant. Antimicrobial susceptibility testing was conducted *in vitro* using the Vitek-2 compact system (bioMérieux, Marcy-l’Etoile, France). Interpretation of antimicrobial susceptibility results followed the Clinical and Laboratory Standards Institute guidelines, which were interpreted according to the European Committee for Antimicrobial Susceptibility Testing criteria (Table S2). Isolates were categorized as susceptible, intermediate, or resistant based on the drug-specific MIC breakpoints outlined in these guidelines. For example, in the case of gentamicin, isolates were classified as susceptible (MIC ≤ 2 mg/L), intermediate (MIC = 4 mg/L), or resistant (MIC ≥ 8 mg/L). Our initial cohort consisted of 88 isolates, with a distribution of 36 susceptible, 1 intermediate, and 51 resistant. To construct a well-defined binary classification problem, the single isolate with an intermediate phenotype was excluded from further analysis. Consequently, the final data set used for developing and validating our predictive models comprised 87 isolates, including 51 (58.6%) resistant and 36 (41.4%) susceptible isolates.

### Preprocessing and sampling

**Fig 1:**
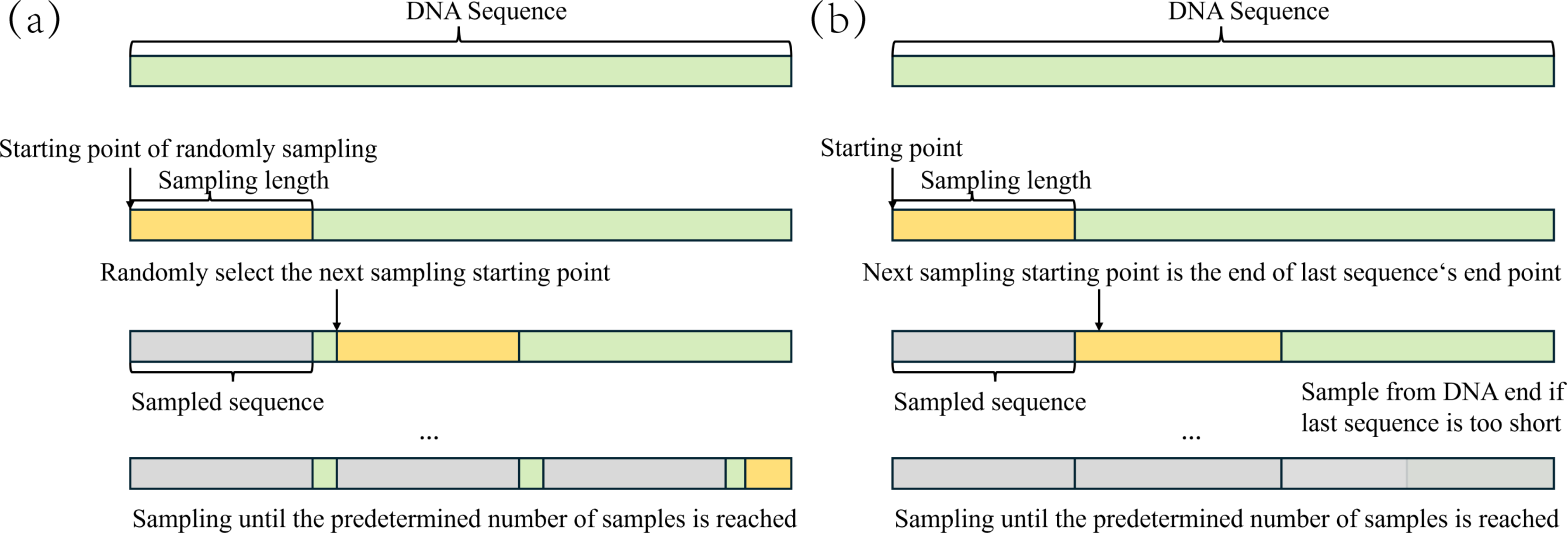
Sampling strategy for training and testing data. The left side illustrates the random sampling approach used in the training data (**a**), where fixed-length segments are randomly extracted from the DNA sequence until the desired number of samples is reached. The right side shows the systematic sampling method for the testing data (**b**), where sequential fixed-length segments are extracted from the start of the sequence, with any shortfall at the end compensated by reverse sampling.

Following initial quality control and masking of whole-genome sequences (as detailed in the Supplemental file and Materials and Methods section), 11 isolates were excluded from the analysis due to failing to meet the predefined quality thresholds, and the final cohort of 87 isolates was partitioned to construct training, validation, and test data sets. To prevent any form of data leakage and ensure a rigorous evaluation of model generalizability, this partitioning was performed strictly at the isolate level, prior to any sequence segmentation. The cohort was split into a training set (*n* = 61), a validation set (*n* = 17), and a test set (*n* = 9), reflecting an approximate 7:2:1 ratio. This isolate-centric partitioning strategy guarantees that all sequence data from a single bacterial isolate are confined to only one dataset split (Fig. 1).

Recent advancements in genomic sequencing technologies have made it necessary to develop sophisticated sampling strategies in order to effectively manage the enormous complexity of genomic data. These strategies encompass systematic sampling, random sampling, adaptive sampling, and stratified sampling, each of which is customized to maximize the utility of genetic data for different analytical purposes (18). Given the extensive length of genetic sequences (approximately 4–6 Mb, for instance, *E. coli*’s genome is around 4.6 Mb), loading entire sequences into the model at once would impose a significant increase in computational demands and necessitate high-performance hardware. To tackle this issue, our study adopts a strategy of segmenting the entire genetic sequence into multiple fragments, thereby reducing the computational load. Initially, we divided the genetic sequences of various bacteria into training, testing, and validation sets in a ratio of 7:2:1. Subsequently, based on the distinct requirements of training and testing, different segmentation approaches were employed.

For the training set, 3,000 fixed-length segments were randomly sampled from each genome. This random sampling approach serves as a form of data augmentation, increasing the diversity of training examples to enhance model robustness and reduce the risk of overfitting. For the validation and test sets, a deterministic, systematic, non-overlapping segmentation approach was employed. Segments were extracted sequentially from the beginning of each genome to ensure complete and unbiased coverage. Fixed-length base segments were extracted sequentially from the 5′ end of each genome. If the final remaining portion was shorter than the predefined segment length, the last segment was instead taken directly from the 3′ terminus of the sequence. All splitting occurred at the isolate (genome) level before any fragment generation, ensuring that no fragments from the same isolate appear in different splits. Training used random segment sampling, whereas validation/test used systematic non-overlapping segments to avoid stochastic bias (Fig. 1). Additionally, the impact of segment length on model performance was also systematically evaluated. This segmentation strategy substantially reduces computational overhead without compromising predictive accuracy, ensuring the method is adaptable to various hardware configurations.

### Development of the deep learning model for gene sequence analysis

Deep learning techniques have been extensively utilized in genomics for tasks such as chromatin accessibility prediction (19), non-coding variant prediction (20), and DNA-protein interaction analysis (21). Convolutional neural networks (CNNs) (22) have been frequently applied in these areas (23) due to their capability of capturing spatial patterns within DNA sequences. Similarly, recurrent neural networks (RNNs), particularly long short-term memory models (24), have been employed for sequence-based genomic tasks, offering advantages in modeling sequential dependencies. However, both CNNs and RNNs exhibit limitations in capturing long-range dependencies across sequences.

To overcome these challenges, we selected a transformer-based architecture. The core rationale for this choice is the transformer’s self-attention mechanism, which is fundamentally designed to capture long-range dependencies across entire sequences. We focus on the architectural rationale: a transformer encoder with dynamic tokenization is suited to long bacterial genomes, capturing long-range dependencies and variable-length motifs more effectively than fixed-token baselines. This design aligns with our empirical gains in whole-sequence prediction accuracy and robustness while maintaining competitive runtime.

### DNA-BERT2 model architecture

**Fig 2:**
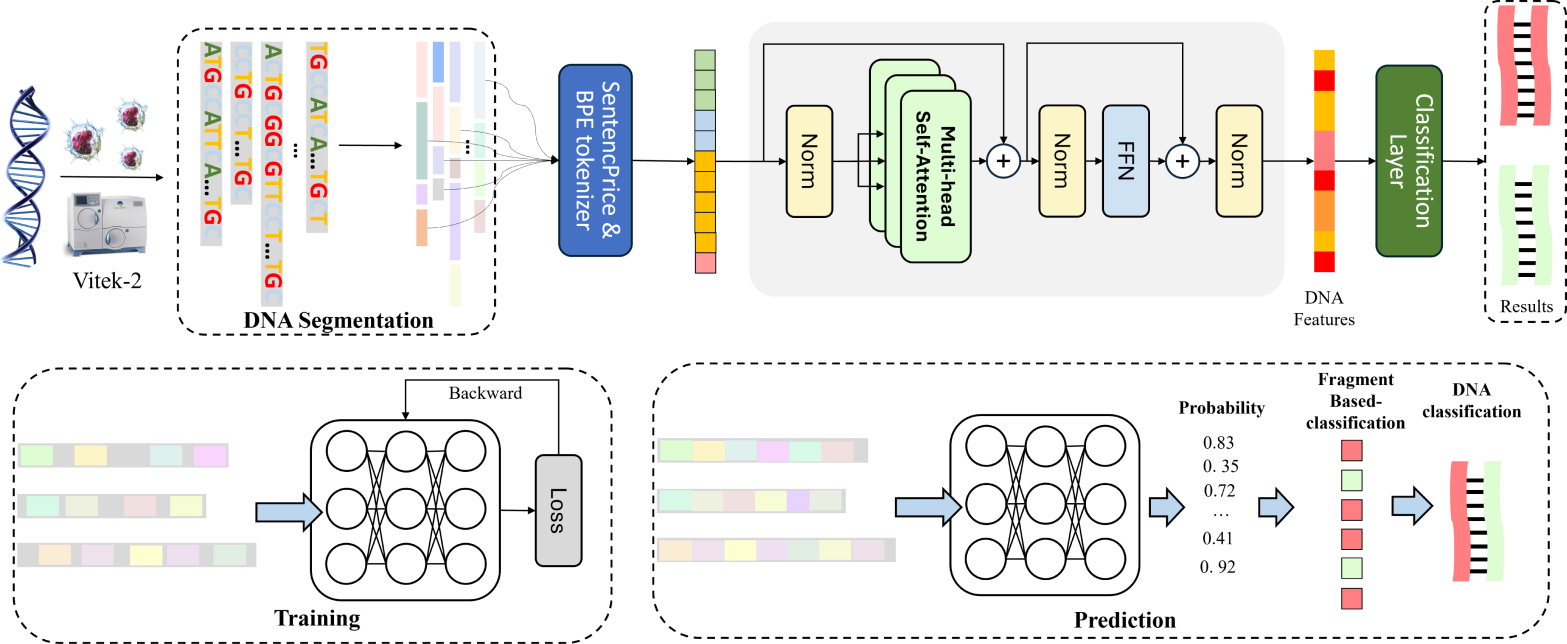
DNA classification workflow, starting from DNA samples processed through a Vitek-2 system. The DNA sequences are segmented and tokenized using SentenPrice & BPE tokenizer, followed by normalization. These tokenized segments are then fed into a transformer model featuring multi-head self-attention and feedforward networks (FFN) for feature extraction. The extracted features are passed to a classification layer to predict DNA classification outcomes. The workflow also includes training and prediction processes, with feedback loops for model optimization, resulting in the classification of DNA fragments. The final output is visualized as a result of DNA classification, distinguishing different classes based on the analyzed DNA features.

The objective of this study was to develop and validate a transformer-based predictor of gentamicin resistance from whole-genome sequences and to prospectively compare dynamic versus fixed tokenization for accuracy, robustness, and runtime. To achieve this, we employed a pre-trained transformer-based foundation model as the core of our methodology. As shown in Fig. 2, transformer models allow for longer DNA sequence inputs, enabling the effective capture of long-range dependencies—an essential feature for accurate genomic predictions (25). For prediction, the DNA sequence is divided into equal-length segments, which are processed sequentially through the tokenizer, transformer layers, and classification layer. By drawing inspiration from DNABERT-2 (26), we utilize SentencePiece (27) with Byte Pair Encoding (BPE) (28) as the tokenizer for DNA segments since it is a language-agnostic tokenizer that treats input as a raw data stream, bypassing the need for pre-tokenization. The tokenized DNA fragments are processed through multiple transformer layers, which leverage multi-head self-attention to capture intricate, long-range dependencies critical for accurate phenotype prediction. Features from each segment are independently passed to a classification layer, and the final prediction for the entire DNA sequence is derived from the aggregated results of all segments. One of the notable strengths of the DNA-BERT2 model is its scalability and adaptability, enabling it to outperform traditional models like CNNs and RNNs by capturing subtle patterns within long and complex genomic sequences.

### Application of DNA-BERT2 in this study

In this research, we fine-tuned a pre-trained DNA-BERT2 model (117 M parameters) for the prediction of gentamicin resistance in *K. pneumoniae* strains based on genomic data. As shown in Fig. 2, the model’s workflow involved segmenting the DNA sequences into fragments of lengths ranging from 200 to 900 base pairs in length to determine the optimal input size. These segments were subsequently tokenized using a SentencePiece with BPE vocabulary. This pre-processing step effectively enabled the model to adapt to different sequence lengths. To enhance computational efficiency and minimize GPU memory requirements, we employed low-rank adaptation (LoRA) for parameter-efficient fine-tuning. The LoRA configuration was set with a rank (r) of 8, an alpha (α) of 16, and a dropout rate of 0.1 applied to the trainable matrices. Fine-tuning was performed on an NVIDIA GeForce RTX 3090 GPU (24 GB RAM) and an 11th Gen Intel Core i9-11900K processor (64 GB RAM) using Python 3.8 and PyTorch (1.13.1). Training was performed with a learning rate of 3e-5, a batch size of 32, and gradient accumulation steps set at 1. The model was trained for 100 epochs, and FP16 precision was enabled to accelerate computation while maintaining model performance. Checkpoints were saved at 200-step intervals, and evaluations were carried out at regular intervals to monitor training progress and optimize the model. Code and an environment file will be provided to enable end-to-end reproduction. This approach enabled us to customize the DNA-BERT2 model for the specific task of predicting gentamicin resistance, thereby significantly enhancing its predictive power and efficiency.

### Model evaluation

Evaluation metrics play a crucial role in assessing the performance of machine learning models and guiding their refinement. In this study, we employed several pivotal performance metrics, comprising accuracy (ACC), precision, recall (Recall), F1-score (F1), and the area under the receiver operating characteristic (ROC) curve (21). These metrics were derived from the fundamental counts of true positives (TP), false negatives (FN), true negatives (TN), and false positives (FP). Accuracy measures the proportion of correctly predicted results throughout the entire data set, offering an overall indicator of model effectiveness. Precision reflects the probability that predicted positive cases are indeed true positives, signifying the model’s reliability in making positive predictions. Recall (sensitivity) assesses the model’s capacity to correctly identify all actual positive instances, ensuring that resistant cases are not missed.

The F1-score, as the harmonic mean of precision and recall, offers a balanced metric, particularly valuable for imbalanced data sets. ROC curves were plotted to compare the true positive rate with the false positive rate at varying thresholds, while AUC summarized the model’s classification performance. Additionally, confusion matrices were used to analyze true and false predictions, providing detailed insights into the model’s strengths and areas for improvement.

## RESULTS

### Performance of fragment-level prediction

**Fig 3:**
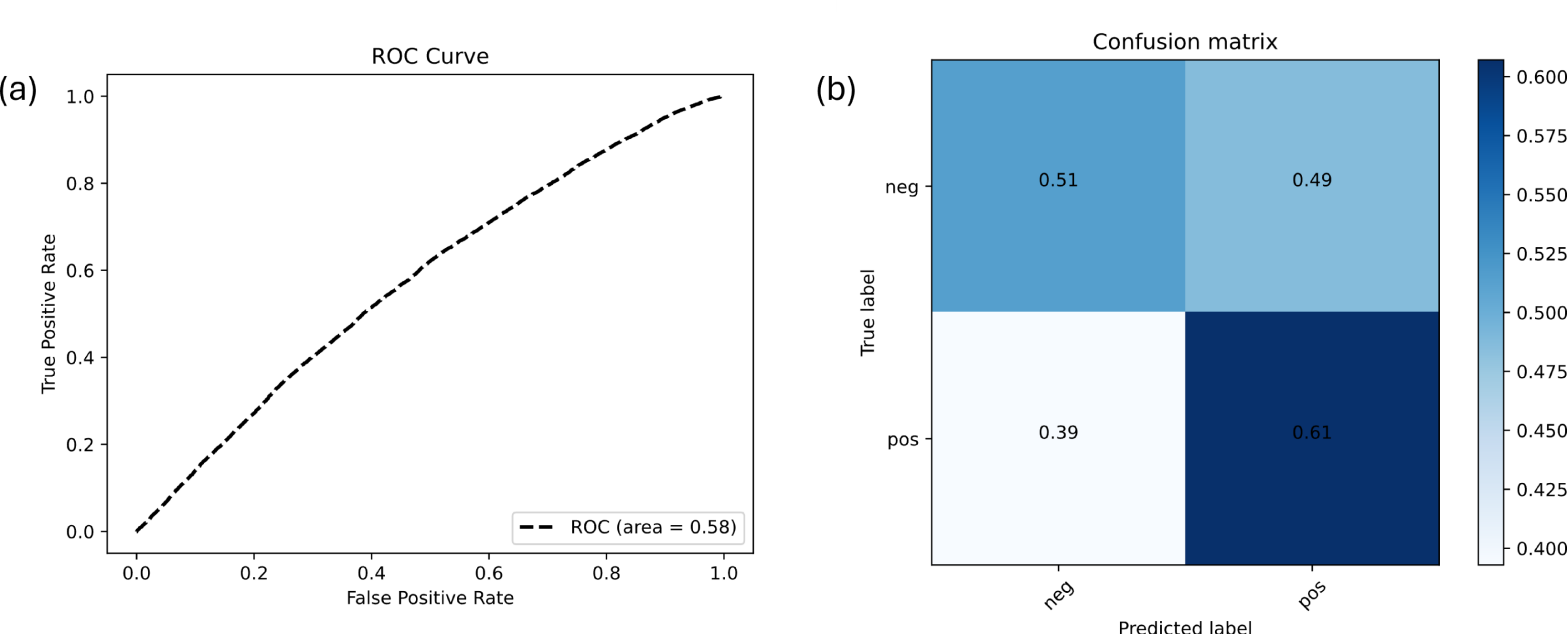
The results of the dynamic tokenizer length model with a 900 sequence length. (**a**) ROC curve of the dynamic tokenizer length model, illustrating the trade-off between true-positive and false-positive rates, with an AUC of 0.58. (**b**) Confusion matrix showing the model’s classification performance across positive and negative labels, with darker colors indicating higher classification proportions.

At the fragment level, the dynamic tokenization strategy consistently outperformed the fixed-tokenization baseline. As shown in Fig. 3, the model’s predictive performance demonstrated a clear upward trend as the sequence length increased. The detailed comparison of fixed and dynamic tokenization strategies for fragment-level resistance prediction is summarized in Table 1. When using a dynamic tokenizer length strategy with a sequence length of 800, the model achieved optimal performance, with an ACC of 66.04% and a recall (REC) of 64.60%. Our findings indicate that models utilizing a dynamic tokenizer length strategy outperformed those using a fixed length strategy, with an accuracy increase of over 6%. Extending the sequence length to 900 base pairs yielded further gains, with precision (PRE) reaching 69.67% and AUC rising to 58%, corresponding to a +6.71% accuracy advantage relative to fixed tokenization. This result suggests that dynamic tokenization is better suited for handling the variability in sequence lengths, allowing the model to make more precise predictions. However, this improvement in accuracy came at the cost of increased prediction time, which grew in proportion to the sequence length. This demonstrates that the dynamic tokenizer length strategy is particularly effective in maximizing the model’s predictive capabilities, especially when working with longer sequences that contain more complex patterns.

**TABLE 1:** The model performance of tokenizer length and different sequence sampling lengths

	Sequence length	ACC (%)	PRE (%)	REC (%)	F1 (%)	Runtime	Samples per second
Fixed tokenizer’s length	200	53.61	26.81	50.00	34.90	14.73	334.04
	250	53.07	26.53	50.00	34.67	14.63	336.07
	300	55.49	54.90	54.24	53.22	14.85	331.27
	350	54.29	55.26	50.14	35.90	14.87	330.95
	400	54.49	27.25	50.00	35.27	15.00	328.10
	450	53.27	26.64	50.00	34.76	14.91	330.01
	500	52.54	51.78	51.59	50.64	14.78	332.90
	550	56.26	56.25	56.29	56.18	14.75	333.60
	600	53.86	53.81	51.86	45.75	15.07	326.54
	650	56.20	58.17	53.66	47.52	14.97	328.70
	700	53.70	26.85	50.00	34.94	14.96	328.83
	750	54.78	56.08	51.22	40.70	14.92	329.72
	800	54.13	55.34	55.10	53.89	14.77	333.14
	850	55.28	55.99	53.49	48.99	14.86	331.19
	900	59.33	59.61	57.74	56.38	15.06	326.72
Dynamic tokenizer’s length	200	62.46	62.81	61.06	60.41	40.16	122.50
	250	63.29	64.30	61.76	60.83	36.82	133.62
	300	53.50	26.75	50.00	34.85	40.74	120.78
	350	63.48	63.60	62.42	62.13	42.21	116.57
	400	64.39	65.71	62.72	61.80	54.71	89.93
	450	64.19	65.86	62.63	61.52	64.22	76.62
	500	63.23	67.16	60.98	58.42	63.47	77.51
	550	63.46	67.01	61.47	59.22	63.32	77.70
	600	64.31	66.06	62.41	61.22	61.56	79.92
	650	65.85	67.57	64.23	63.39	75.46	65.20
	700	57.83	58.01	58.03	57.82	85.71	57.41
	750	63.62	63.41	62.94	62.91	164.51	14.95
	800	66.04	68.64	64.60	63.44	479.65	10.26
	850	65.49	68.87	63.80	62.21	544.52	9.04
	900	65.22	69.67	63.53	61.48	509.60	9.66

However, the modest AUC value (0.58) highlights the inherent limitations of predicting phenotype from short DNA fragments. These segments often contain only partial genetic information and lack the broader genomic context required to confidently discriminate between resistant and susceptible isolates. While adaptive tokenization improved the extraction of local sequence information, the predictive power remained fundamentally constrained by the limited information density within these short inputs.

A statistical comparison of the two tokenization strategies at the fragment level is provided in Table 2, which reports the mean performance differences, bootstrap-based 95% confidence intervals, and paired significance tests (*t*-test and Wilcoxon). These results quantitatively confirm that the dynamic tokenizer offers statistically significant improvements in ACC, PRE, REC, and F1 over the fixed-length tokenizer.

**TABLE 2:** Statistical comparison of dynamic versus fixed tokenization strategies at the fragment level

Metric	Mean difference (pct)	95% CI (bootstrap)	Paired *t*-test *P* value	Wilcoxon *P* value
ACC	+8.4	(+5.1, +10.8)	2.4 × 10⁻⁷	1.2 × 10⁻⁴
PRE	+16.9	(+10.0, +23.9)	0.0023	0.0026
REC	+9.2	(+5.1, +13.3)	1.1 × 10⁻⁶	1.2 × 10⁻⁴
F1	+15.2	(+7.8, +22.7)	3.3 × 10⁻⁴	0.0015

Overall, our results highlight the superior performance of the dynamic tokenizer length strategy, especially with longer sequence lengths. While the increased prediction time remains a consideration, the significant gains in accuracy and other performance metrics validate the dynamic tokenizer strategy as an effective approach for improving deep learning-based genomic prediction models. This insight is particularly valuable in the context of microbial resistance prediction, where capturing intricate sequence patterns is crucial for accurate model performance.

### Performance of full-sequence resistance prediction

The primary goal of this study was to assess the ability of different models to predict bacterial resistance based on gene fragment sequences, ultimately using these predictions to evaluate resistance for the entire gene sequence. Table 3 compares the performance of models employing fixed and dynamic tokenizer lengths for resistance prediction based on full sequences. The results clearly show that the dynamic tokenizer models achieved 76.47% accuracy, an F1 score of 83.33%, and an AUC of 0.77. This represents a substantial improvement over the fixed-tokenization baseline, which recorded a consistent accuracy of 64.71%, translating to a +11.76% increase in accuracy and a +4.1% increase in F1 score. This suggests that the fixed tokenizer strategy, while producing stable results, does not fully capture the complexity of the gene sequences, leading to limited predictive performance. The lack of flexibility in handling variable-length gene sequences may explain the lower accuracy observed with the fixed tokenizer approach.

**TABLE 3:** The model performance based on whole-sequence prediction

	Sequence length	ACC (%)	PRE (%)	REC (%)	F1 (%)	Per-sample time (s)
Fixed tokenizer’s length	200	64.71	64.71	100.00	78.57	1.2
	250	64.71	64.71	100.00	78.57	2.5
	300	64.71	64.71	100.00	78.57	3.8
	350	64.71	64.71	100.00	78.57	4.0
	400	64.71	64.71	100.00	78.57	4.5
	450	64.71	64.71	100.00	78.57	4.3
	500	64.71	64.71	100.00	78.57	4.4
	550	64.71	64.71	100.00	78.57	5.0
	600	64.71	64.71	100.00	78.57	5.7
	650	64.71	64.71	100.00	78.57	6.2
	700	64.71	64.71	100.00	78.57	5.3
	750	64.71	64.71	100.00	78.57	5.5
	800	64.71	64.71	100.00	78.57	5.0
	850	64.71	64.71	100.00	78.57	5.2
	900	64.71	64.71	100.00	78.57	5.7
Dynamic tokenizer’s length	200	76.47	76.92	90.91	83.33	1.5
	250	76.47	76.92	90.91	83.33	3.0
	300	64.71	64.71	100.00	78.57	3.3
	350	64.71	64.71	100.00	78.57	4.6
	400	70.59	80.00	72.73	76.19	5.0
	450	70.59	80.00	72.73	76.19	5.9
	500	76.47	76.92	90.91	83.33	6.2
	550	64.71	77.78	63.64	70.00	7.0
	600	70.59	80.00	72.73	76.19	6.7
	650	70.59	80.00	72.73	76.19	7.3
	700	64.71	64.71	100.00	78.57	6.6
	750	76.47	76.92	90.91	83.33	6.7
	800	76.47	76.92	90.91	83.33	6.2
	850	70.59	80.00	72.73	76.19	5.9
	900	64.71	64.71	100.00	78.57	6.1

**Fig 4:**
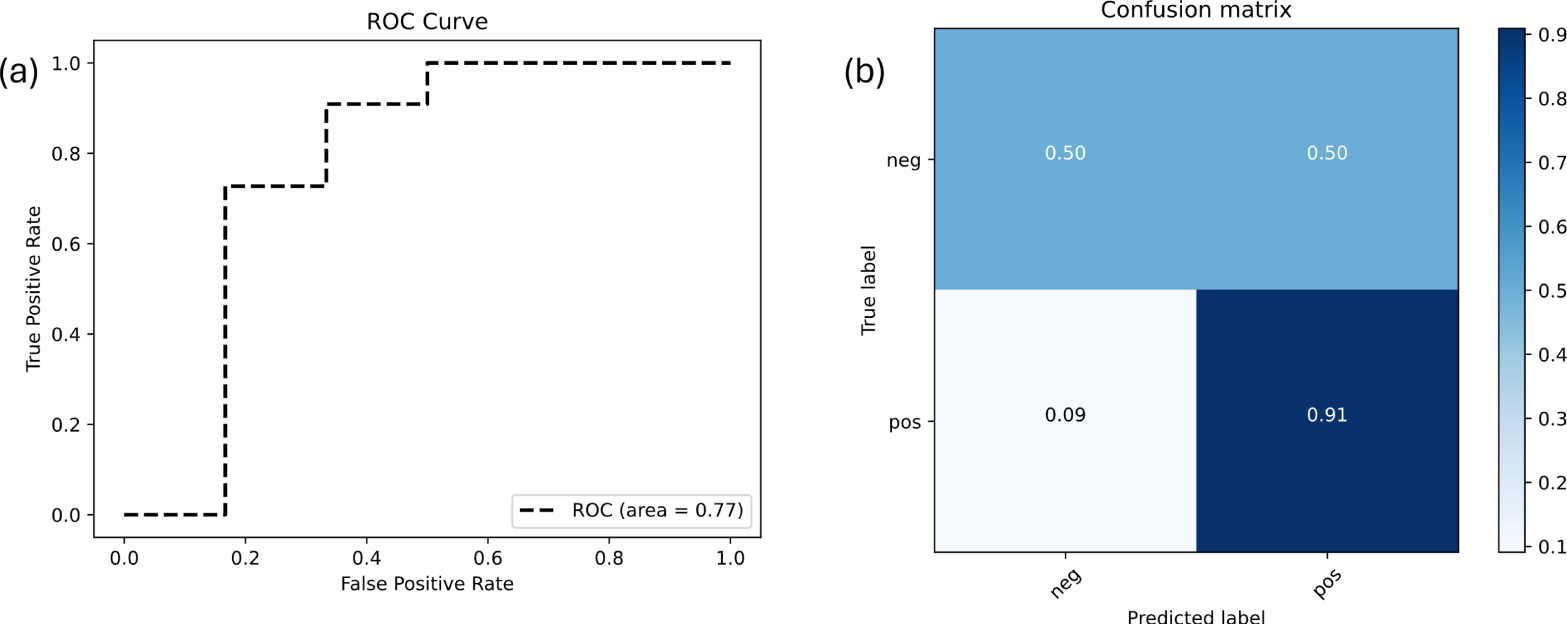
The results of the dynamic tokenizer length model with 900 sequence length in whole-sequence prediction. (**a**) ROC curve of the dynamic tokenizer length model, demonstrating its ability to distinguish positive and negative samples with an AUC of 0.77, indicating a substantial improvement compared with fragment-level predictions. (**b**) Confusion matrix illustrating the classification outcomes for positive and negative samples, where the model achieved a notably higher true positive rate (0.91) while maintaining moderate balance in negative class recognition. Darker shades correspond to higher prediction accuracy across respective categories.

Further analysis of the dynamic tokenizer model revealed that the optimal performance was achieved when the sampling fragment length was set to 900. As shown in the predictive ROC curve and confusion matrix in Fig. 4, this model yielded an area under the curve (21) of 77%, demonstrating its strong ability to predict antibiotic resistance with high accuracy. The ROC curve and confusion matrix further illustrate that the dynamic tokenizer model, particularly with a sequence length of 900, was able to generate more reliable predictions, outperforming the fixed tokenizer models in both sensitivity and specificity.

A statistical comparison of dynamic versus fixed tokenization strategies in full-sequence prediction is provided in Table 4. Unlike the fragment-level results, the full-sequence evaluation shows a more nuanced performance pattern. Dynamic tokenization delivers significantly higher accuracy and precision, with mean improvements of +5.88 and +10.04 percentage points, respectively, both supported by narrow bootstrap confidence intervals and strong significance in paired *t*-tests and Wilcoxon tests. Under the fixed tokenization strategy, the model exhibited a strong bias toward predicting the majority class, which artificially inflated recall by assigning most samples to the dominant category. Once this bias was mitigated by the dynamic tokenization strategy—which provides more discriminative capacity and reduces overfitting to the majority class—the recall naturally decreased to a more realistic level. Taken together, these results highlight the dynamic tokenization strategy as a more robust and effective modeling choice.

**TABLE 4:** Statistical comparison of dynamic versus fixed tokenization strategies in full-sequence prediction

Metric	Mean difference (pct)	95% CI (bootstrap)	Paired *t*-test *P* value	Wilcoxon *P* value
ACC	+5.88	(+3.53, +8.23)	4.3 × 10⁻⁴	0.0040
PRE	+10.04	(+6.77, +12.95)	2.9 × 10⁻⁵	0.0028
REC	−14.54	(−20.60, −8.48)	5.9 × 10⁻⁴	0.0028
F1	+0.22	(−1.71, +2.06)	0.829	0.525

The marked improvement in AUC from fragment-level (0.58) to full-sequence (0.77) underscores the importance of broader sequence context. Longer sequences provide richer structural and functional motifs, enabling the model to detect resistance-associated patterns that are absent or diluted in short fragments. Dynamic tokenization further amplified this advantage by flexibly adapting to local variability across the full gene, leading to more reliable sensitivity and specificity (Fig. 4). Together, these findings suggest that while fragment-level modeling offers efficiency and partial predictive value, robust resistance prediction requires full-sequence analysis, where dynamic tokenization demonstrates clear superiority over fixed strategies.

## DISCUSSION

### Key findings

This research has developed a sophisticated approach for predicting bacterial gentamicin resistance in *K. pneumoniae*. The principal finding is that a dynamic tokenization strategy is significantly superior to a fixed-tokenization approach for this task. By adapting to the sequence variability inherent in bacterial genomes, our model achieved a full-sequence prediction accuracy of 76.47% and an F1 score of 83.33%, representing a substantial +11.76% improvement in accuracy over the fixed-tokenization baseline. This demonstrates that architectural choices at the tokenization level are critical for unlocking the full potential of transformer models in genomics. The model’s ability to effectively process longer DNA segments and capture complex, long-range dependencies is fundamental to this performance gain.

### Comparison with existing work

Our approach advances the field of AMR prediction beyond traditional deep learning architectures and recent specialized models. Unlike conventional CNNs, which are constrained by local receptive fields, or RNNs, which struggle with the long sequences typical of genomes (23), our transformer-based model efficiently handles whole-sequence data. Our results demonstrate substantial improvements in comparison to recent deep-learning models for antibiotic resistance prediction. Ren et al. (29) used a transfer-learning-based approach for novel antibiotics, attaining robustness but having limited adaptability to different sequence lengths, which is overcome by our dynamic tokenization method. Weis et al. (30) applied machine learning to MALDI-TOF mass spectrometry data with high accuracy, yet their method’s dependence on mass spectrometry restricts scalability. In contrast, our DNA-BERT2 model, which is based on genomic data, is more flexible across bacterial species. Moreover, DeepARG (31) focused on metagenomic data, but our model provides a more accurate solution for clinical isolates, handling both short and long sequences with higher consistency, owing to its multi-head self-attention mechanism and dynamic tokenization.

### Clinical implications and future translational workflow

In the workflow validated here, which utilizes standard short-read WGS from a cultured isolate, the upstream biological processes remain the primary rate-limiting steps. The total time from a bacterial colony to a final resistance prediction is estimated at 24–36 h (comprising DNA extraction: ~2–4 h; library preparation: ~4–8 h; sequencing run: ~12–24 h). In stark contrast, the computational inference time for our model, once the FASTA sequence is generated, is negligible (<1 h on a single GPU). While this 24–36 h timeline does not yet offer a transformative speed advantage over conventional phenotypic AST for cultured isolates, the principal value of our approach lies in its future compatibility with rapid sequencing technologies. The true translational potential will be unlocked as these platforms mature. For instance, emerging long-read sequencing workflows paired with rapid library preparation kits could foreseeably reduce the isolate-to-result timeframe to a clinically actionable 8–12 h. Furthermore, integration with direct-from-specimen metagenomic sequencing could eventually obviate the need for culture altogether, representing the ultimate goal for rapid, targeted diagnostics. Direct-from-specimen prediction is feasible but requires addressing host/background DNA and species mixing. A pragmatic path is (i) shotgun metagenomics with taxonomic binning; (ii) species-specific contig/read assignment; (iii) applying our genome-sequence predictor to binned pathogen material. Monomicrobial positive blood cultures are a near-term translational scenario; polymicrobial sputum will additionally require read-level deconvolution.

### Limitations and future directions

Despite the promising outcomes, this study is not without its limitations: (i) the current model was trained and validated on a data set of a single species (*K. pneumoniae*) for a single antibiotic (gentamicin). Its performance is not yet proven for other pathogens or drug classes. Future work must incorporate larger, multi-center data sets representing diverse geographic locations and strain types to ensure generalizability. (ii) As with many deep learning models, our transformer architecture functions as a “black box.” A significant future step is to enhance model interpretability by implementing attribution techniques to identify the specific genomic loci (e.g., resistance genes, mutations, or regulatory elements) that most influence a resistance prediction. This is essential for building clinical trust and potentially uncovering novel resistance mechanisms. (iii) The improved accuracy of dynamic tokenization and long-sequence analysis comes at the cost of increased computational demand. This may present a barrier to implementation in resource-limited clinical or laboratory settings.

The current model is trained on binary phenotypes (resistant vs susceptible) and does not output mechanistic labels. We plan a mechanism-aware extension by integrating curated AMR annotations (e.g., aminoglycoside-modifying enzymes, 16S rRNA methyltransferases, efflux) and adding a multi-label head alongside the phenotype head, with token/region-level attribution to link predictions to genomic loci. Notably, mechanism prediction requires high-quality gene-level labels, which were not available for our study cohort, and this will be a key focus of our follow-up work.

### Conclusion

This study presents a robust framework for predicting bacterial resistance from genomic sequences using deep learning. By introducing a dynamic tokenizer length strategy, our model achieved accuracy improvements of over 6% at the fragment level and +11.76% accuracy with +4.76% F1 gains in full-sequence resistance prediction compared to fixed-length tokenizers. These gains were further reflected in the AUC, which increased from 0.58 at the fragment level to 0.77 in full-sequence prediction, highlighting the importance of broader sequence context for robust discrimination. This approach represents a critical step toward the development of rapid, sequence-based diagnostics that could enable more timely and precise therapeutic decisions, ultimately aiding in the global effort to combat antimicrobial resistance. Future research should focus on expanding the applicability of these methods to other forms of resistance and further refining the model to ensure its utility in diverse clinical and laboratory settings. Ultimately, by enabling faster and more accurate prediction of antimicrobial resistance directly from genomic data, this approach has the potential to shorten diagnostic turnaround times, support timely therapeutic decision-making, and improve clinical outcomes in the fight against drug-resistant infections.

## SUPPLEMENTAL MATERIAL

Table S1 spectrum.01906-25-s0001.xlsx Summary of clinical *K. pneumoniae* isolates used in this study.

Table S2 spectrum.01906-25-s0002.docx Antimicrobial susceptibility testing profiles for the *K. pneumoniae* isolates.

ASM does not own the copyrights to Supplemental Material that may be linked to, or accessed through, an article. The authors have granted ASM a non-exclusive, world-wide license to publish the Supplemental Material files. Please contact the corresponding author directly for reuse.
