# Supplementary material for: Construction and validation of a phenotypic prediction model for bacterial gentamicin resistance using deep learning with gene sequences
Source: Microbiol Spectr. 2026 Jul 15;14(8):e01906-25. doi: 10.1128/spectrum.01906-25 (PMC13436402; doi:10.1128/spectrum.01906-25)
Supplement: Supplementary file 1 [file spectrum.01906-25-s0002.docx]

**Table S2. Antimicrobial susceptibility testing profiles for the *K. pneumoniae* isolates.** Minimal inhibitory concentrations (MICs) were determined for 15 antimicrobial agents against 98 isolates. All MIC values are reported in micrograms per milliliter (μg/mL). The interpretation of MICs as Susceptible (S), Intermediate (I), or Resistant (R) was based on the Clinical and Laboratory Standards Institute (CLSI) 2024 M100 guidelines. Abbreviations and symbols are as follows: ≥, MIC is greater than or equal to the highest concentration tested; ≤, MIC is less than or equal to the lowest concentration tested; NA, result not available or test not performed. Antibiotic Abbreviations: IPM, Imipenem; MEM, Meropenem; CAZ, Ceftazidime; FEP, Cefepime; ATM, Aztreonam; AMK, Amikacin; CIP, Ciprofloxacin; LVX, Levofloxacin; PIPC, Piperacillin/Tazobactam; CPS, Cefoperazone/Sulbactam; PB, Polymyxin; TET, Tetracycline; SXT, Cotrimoxazole; GEN, Gentamicin; TOB, Tobramycin.

| id | Strains | IPM | MEM | CAZ | FEP | ATM | AMK | CIP | LVX | TZP | CPS | PB | TET | SXT | GEN | TOB |
| --- | --- | --- | --- | --- | --- | --- | --- | --- | --- | --- | --- | --- | --- | --- | --- | --- |
| 1 | xz109 | ≥16 | ≥16 | ≥64 | ≥64 | ≥64 | ≥64 | ≥4 | ≥8 | ≥128 | ≥64 | ≤0.5 | ≤0.5 | ≤20 | ≥16 | ≥16 |
| 2 | xz125 | ≥16 | ≥16 | ≥64 | ≥64 | ≥64 | ≥64 | ≥4 | ≥8 | ≥128 | ≥64 | ≤0.5 | ≤0.5 | ≤20 | ≥16 | ≥16 |
| 3 | xz081 | ≥16 | ≥16 | ≥64 | ≥32 | ≥64 | ≥64 | ≥4 | ≥8 | ≥128 | ≥64 | ≤0.5 | ≤0.5 | ≤20 | ≥16 | ≥16 |
| 4 | xz155 | ≥16 | ≥16 | ≥64 | ≥64 | ≥64 | ≥64 | ≥4 | ≥8 | ≥128 | ≥64 | ≤0.5 | ≤0.5 | ≤20 | ≥16 | ≥16 |
| 5 | xz143 | ≥16 | ≥16 | ≥64 | ≥64 | ≥64 | ≥64 | ≥4 | ≥8 | ≥128 | ≥64 | ≤0.5 | ≤0.5 | ≤20 | ≥16 | ≥16 |
| 6 | xz127 | ≥16 | ≥16 | ≥64 | ≥64 | ≥64 | ≥64 | ≥4 | ≥8 | ≥128 | ≥64 | ≤0.5 | ≤0.5 | ≤20 | 4 | ≥16 |
| 7 | xz074 | ≥16 | ≥16 | ≥64 | ≥32 | ≥64 | ≥64 | ≥4 | ≥8 | ≥128 | ≥64 | ≤0.5 | ≤0.5 | ≤20 | ≥16 | ≥16 |
| 8 | xz117 | ≥16 | ≥16 | ≥64 | ≥64 | ≥64 | ≤2 | ≥4 | ≥8 | ≥128 | ≥64 | ≤0.5 | ≤0.5 | ≤20 | ≤1 | ≤1 |
| 9 | xz115 | ≥16 | ≥16 | ≥64 | ≥64 | ≥64 | ≤2 | ≥4 | ≥8 | ≥128 | ≥64 | ≤0.5 | ≤0.5 | ≤20 | ≤1 | ≤1 |
| 10 | xz066 | ≥16 | ≥16 | ≥64 | ≥32 | ≥64 | ≥64 | ≥4 | ≥8 | ≥128 | ≥64 | ≤0.5 | ≤0.5 | ≤20 | ≥16 | ≥16 |
| 11 | xz079 | ≥16 | ≥16 | ≥64 | ≥32 | ≥64 | ≥64 | ≥4 | ≥8 | ≥128 | ≥64 | ≤0.5 | ≤0.5 | ≤20 | ≥16 | ≥16 |
| 12 | xz071 | ≥16 | ≥16 | ≥64 | ≥32 | ≥64 | ≥64 | ≥4 | ≥8 | ≥128 | ≥64 | ≤0.5 | ≤0.5 | ≤20 | ≥16 | ≥16 |
| 13 | xz141 | ≥16 | ≥16 | ≥64 | ≥64 | ≥64 | ≥64 | ≥4 | ≥8 | ≥128 | ≥64 | ≤0.5 | ≤0.5 | ≤20 | ≥16 | ≥16 |
| 14 | xz142 | ≥16 | ≥16 | ≥64 | ≥64 | ≥64 | ≥64 | ≥4 | ≥8 | ≥128 | ≥64 | ≤0.5 | ≤0.5 | ≤20 | ≥16 | ≥16 |
| 15 | xz166 | ≥16 | ≥16 | ≥64 | ≥64 | ≥64 | ≤2 | ≥4 | ≥8 | ≥128 | ≥64 | ≤0.5 | ≤0.5 | ≤20 | ≤1 | ≤1 |
| 16 | xz112 | ≥16 | ≥16 | ≥64 | ≥64 | ≥64 | ≥64 | ≥4 | ≥8 | ≥128 | ≥64 | ≤0.5 | ≤0.5 | ≤20 | ≥16 | ≥16 |
| 17 | xz157 | ≥16 | ≥16 | ≥64 | ≥64 | ≥64 | ≤2 | ≥4 | ≥8 | ≥128 | ≥64 | ≤0.5 | ≤0.5 | ≥320 | ≤1 | ≤1 |
| 18 | xz146 | ≥16 | ≥16 | ≥64 | ≥64 | ≥64 | ≤2 | ≥4 | ≥8 | ≥128 | ≥64 | ≤0.5 | ≤0.5 | ≤20 | ≤1 | ≤1 |
| 19 | xz135 | ≥16 | ≥16 | ≥64 | ≥64 | ≥64 | ≤2 | ≥4 | ≥8 | ≥128 | ≥64 | ≤0.5 | ≤0.5 | ≤20 | ≤1 | ≤1 |
| 20 | xz119 | ≥16 | ≥16 | ≥64 | ≥64 | ≥64 | ≤2 | ≥4 | ≥8 | ≥128 | ≥64 | ≤0.5 | ≤0.5 | ≤20 | ≤1 | ≤1 |
| 21 | xz126 | ≥16 | ≥16 | ≥64 | ≥64 | ≥64 | ≥64 | ≥4 | ≥8 | ≥128 | ≥64 | ≤0.5 | ≤0.5 | ≤20 | ≥16 | ≥16 |
| 22 | xz092 | ≥16 | ≥16 | ≥64 | ≥32 | ≥64 | ≥64 | ≥4 | ≥8 | ≥128 | ≥64 | ≤0.5 | ≤0.5 | ≤20 | ≥16 | ≥16 |
| 23 | xz110 | ≥16 | ≥16 | ≥64 | ≥64 | ≥64 | ≥64 | ≥4 | ≥8 | ≥128 | ≥64 | ≤0.5 | ≤0.5 | ≤20 | ≥16 | ≥16 |
| 24 | xz138 | ≥16 | ≥16 | ≥64 | ≥64 | ≥64 | ≥64 | ≥4 | ≥8 | ≥128 | ≥64 | ≤0.5 | ≤0.5 | ≤20 | ≥16 | ≥16 |
| 25 | xz178 | ≥16 | ≥16 | ≥64 | ≥64 | ≥64 | ≥64 | ≥4 | ≥8 | ≥128 | ≥64 | ≤0.5 | 1 | ≤20 | ≥16 | ≥16 |
| 26 | xz123 | ≥16 | ≥16 | ≥64 | ≥64 | ≥64 | ≥64 | ≥4 | ≥8 | ≥128 | ≥64 | ≤0.5 | ≤0.5 | ≤20 | ≥16 | ≥16 |
| 27 | xz158 | ≥16 | ≥16 | ≥64 | ≥64 | ≥64 | ≥64 | ≥4 | ≥8 | ≥128 | ≥64 | ≤0.5 | ≤0.5 | ≤20 | ≥16 | ≥16 |
| 28 | xz167 | ≥16 | ≥16 | ≥64 | ≥64 | ≥64 | ≥64 | ≥4 | ≥8 | ≥128 | ≥64 | ≤0.5 | ≤0.5 | ≤20 | ≥16 | ≥16 |
| 29 | xz083 | ≥16 | ≥16 | ≥64 | ≥32 | ≥64 | ≥64 | ≥4 | ≥8 | ≥128 | ≥64 | ≤0.5 | ≤0.5 | ≤20 | ≥16 | ≥16 |
| 30 | xz116 | ≥16 | ≥16 | ≥64 | ≥64 | ≥64 | ≥64 | ≥4 | ≥8 | ≥128 | ≥64 | ≤0.5 | ≤0.5 | ≤20 | ≥16 | ≥16 |
| 31 | xz072 | ≥16 | ≥16 | ≥64 | ≥32 | ≥64 | ≤2 | ≥4 | ≥8 | ≥128 | ≥64 | ≤0.5 | ≤0.5 | ≤20 | ≥16 | ≤1 |
| 32 | xz121 | ≥16 | ≥16 | ≥64 | ≥64 | ≥64 | ≥64 | ≥4 | ≥8 | ≥128 | ≥64 | ≤0.5 | ≤0.5 | ≤20 | ≥16 | ≥16 |
| 33 | xz133 | ≥16 | ≥16 | ≥64 | ≥64 | ≥64 | ≥64 | ≥4 | ≥8 | ≥128 | ≥64 | ≤0.5 | ≤0.5 | ≤20 | ≥16 | ≥16 |
| 34 | xz150 | ≥16 | ≥16 | ≥64 | ≥64 | ≥64 | ≥64 | ≥4 | ≥8 | ≥128 | ≥64 | ≤0.5 | ≤0.5 | ≤20 | ≥16 | ≥16 |
| 35 | xz131 | ≥16 | ≥16 | ≥64 | ≥64 | ≥64 | ≥64 | ≥4 | ≥8 | ≥128 | ≥64 | ≤0.5 | ≤0.5 | ≤20 | ≥16 | ≥16 |
| 36 | xz139 | ≥16 | ≥16 | ≥64 | ≥64 | ≥64 | ≥64 | ≥4 | ≥8 | ≥128 | ≥64 | ≤0.5 | ≤0.5 | ≤20 | ≥16 | ≥16 |
| 37 | xz107 | ≥16 | ≥16 | ≥64 | ≥64 | ≥64 | ≥64 | ≥4 | ≥8 | ≥128 | ≥64 | ≤0.5 | ≤0.5 | ≤20 | ≥16 | ≥16 |
| 38 | xz090 | ≥16 | ≥16 | ≥64 | ≥32 | ≥64 | ≥64 | ≥4 | ≥8 | ≥128 | ≥64 | ≤0.5 | ≤0.5 | ≤20 | ≥16 | ≥16 |
| 39 | xz163 | ≥16 | ≥16 | ≥64 | ≥64 | ≥64 | ≥64 | ≥4 | ≥8 | ≥128 | ≥64 | ≤0.5 | ≤0.5 | ≥320 | ≥16 | ≥16 |
| 40 | xz168 | ≥16 | ≥16 | ≥64 | ≥64 | ≥64 | ≥64 | ≥4 | ≥8 | ≥128 | ≥64 | ≤0.5 | ≤0.5 | ≤20 | ≥16 | ≥16 |
| 41 | xz181 | ≥16 | ≥16 | ≥64 | ≥64 | ≥64 | ≥64 | ≥4 | ≥8 | ≥128 | ≥64 | ≤0.5 | ≤0.5 | ≤20 | ≥16 | ≥16 |
| 42 | xz061 | ≥16 | ≥16 | ≥64 | ≥32 | ≥64 | ≥64 | ≥4 | ≥8 | ≥128 | ≥64 | ≤0.5 | ≤0.5 | ≤20 | ≥16 | ≥16 |
| 43 | xz128 | ≥16 | ≥16 | ≥64 | ≥64 | ≥64 | ≥64 | ≥4 | ≥8 | ≥128 | ≥64 | ≤0.5 | ≤0.5 | ≤20 | ≥16 | ≥16 |
| 44 | xz059 | ≥16 | ≥16 | ≥64 | ≥32 | ≥64 | ≥64 | ≥4 | ≥8 | ≥128 | ≥64 | ≤0.5 | ≤0.5 | ≤20 | ≥16 | ≥16 |
| 45 | xz065 | ≥16 | ≥16 | ≥64 | ≥32 | ≥64 | ≥64 | ≥4 | ≥8 | ≥128 | ≥64 | ≤0.5 | ≤0.5 | ≤20 | ≥16 | ≥16 |
| 46 | xz124 | ≥16 | ≥16 | ≥64 | ≥64 | ≥64 | ≥64 | ≥4 | ≥8 | ≥128 | ≥64 | ≤0.5 | ≤0.5 | ≤20 | ≥16 | ≥16 |
| 47 | xz106 | ≥16 | ≥16 | ≥64 | ≥64 | ≥64 | ≥64 | ≥4 | ≥8 | ≥128 | ≥64 | ≤0.5 | ≤0.5 | ≤20 | ≥16 | ≥16 |
| 48 | xz148 | ≥16 | ≥16 | ≥64 | ≥64 | ≥64 | ≤2 | 0.5 | 4 | ≥128 | ≥64 | ≤0.5 | ≤0.5 | ≤20 | ≤1 | ≤1 |
| 49 | xz160 | ≥16 | ≥16 | ≥64 | ≥64 | ≥64 | ≤2 | ≥4 | ≥8 | ≥128 | ≥64 | ≤0.5 | ≤0.5 | ≤20 | ≤1 | ≤1 |
| 50 | xz183 | ≥16 | ≥16 | ≥64 | ≥64 | ≥64 | ≥64 | ≥4 | ≥8 | ≥128 | ≥64 | ≤0.5 | 1 | ≤20 | ≥16 | ≥16 |
| 51 | xz140 | ≥16 | ≥16 | ≥64 | ≥64 | ≥64 | ≥64 | ≥4 | ≥8 | ≥128 | ≥64 | ≤0.5 | ≤0.5 | ≤20 | ≥16 | ≥16 |
| 52 | xz147 | ≥16 | ≥16 | ≥64 | ≥64 | ≥64 | ≥4 | ≥4 | ≥8 | ≥128 | ≥64 | ≤0.5 | ≤0.5 | ≤20 | ≥16 | ≥16 |
| 53 | xz156 | ≥16 | ≥16 | ≥64 | ≥64 | ≥64 | ≥64 | ≥4 | ≥8 | ≥128 | ≥64 | ≤0.5 | ≤0.5 | ≤20 | ≥16 | ≥16 |
| 54 | xz161 | ≥16 | ≥16 | ≥64 | ≥64 | ≥64 | ≥64 | ≥4 | ≥8 | ≥128 | ≥64 | ≤0.5 | ≤0.5 | ≤20 | ≥16 | ≥16 |
| 55 | xz159 | ≥16 | ≥16 | ≥64 | ≥64 | ≥64 | ≥64 | ≥4 | ≥8 | ≥128 | ≥64 | ≤0.5 | ≤0.5 | ≤20 | ≤1 | ≥16 |
| 56 | xz069 | ≥16 | ≥16 | ≥64 | ≥32 | ≥64 | ≤2 | ≥4 | ≥8 | ≥128 | ≥64 | ≤0.5 | ≤0.5 | ≤20 | ≤1 | ≤1 |
| 57 | xz154 | ≥16 | ≥16 | ≥64 | ≥64 | ≥64 | ≤2 | ≥4 | ≥8 | ≥128 | ≥64 | ≤0.5 | ≤0.5 | ≤20 | ≤1 | ≤1 |
| 58 | xz179 | ≥16 | ≥16 | ≥64 | 4 | ≥64 | ≥64 | ≥4 | ≥8 | ≥128 | ≥64 | ≤0.5 | 1 | ≤20 | ≥16 | ≥16 |
| 59 | xz137 | ≥16 | ≥16 | ≥64 | ≥64 | ≥64 | ≥64 | ≥4 | ≥8 | ≥128 | ≥64 | ≤0.5 | ≤0.5 | ≤20 | ≥16 | ≥16 |
| 60 | xz169 | ≥16 | ≥16 | ≥32 | ≥64 | ≥64 | ≥64 | ≥4 | ≥8 | ≥128 | ≥64 | ≤0.5 | ≤0.5 | ≤20 | ≥16 | ≥16 |
| 61 | xz080 | ≥16 | ≥16 | ≥64 | ≥32 | ≥64 | ≥64 | ≥4 | ≥8 | ≥128 | ≥64 | ≤0.5 | ≤0.5 | ≤20 | ≥16 | ≥16 |
| 62 | xz144 | ≥16 | ≥16 | ≥64 | ≥64 | ≥64 | ≤2 | ≥4 | ≥8 | ≥128 | ≥64 | ≤0.5 | ≤0.5 | ≤20 | ≤1 | ≤1 |
| 63 | xz184 | ≥16 | ≥16 | ≥64 | ≥64 | ≥64 | ≥64 | ≥4 | ≥8 | ≥128 | ≥64 | ≤0.5 | 1 | ≤20 | ≥16 | ≥16 |
| 64 | xz180 | ≥16 | ≥16 | ≥64 | ≥64 | ≥64 | ≥64 | ≥4 | ≥8 | ≥128 | ≥64 | ≤0.5 | 2 | ≤20 | ≥16 | ≥16 |
| 65 | xz152 | ≥16 | ≥16 | ≥64 | ≥64 | ≥64 | ≥64 | ≥4 | ≥8 | ≥128 | ≥64 | ≤0.5 | ≤0.5 | ≥320 | ≥16 | ≥16 |
| 66 | xz171 | ≥16 | ≥16 | ≥64 | ≥64 | ≥64 | ≥64 | ≥4 | ≥8 | ≥128 | ≥64 | ≤0.5 | ≤0.5 | ≥320 | ≥16 | ≥16 |
| 67 | xz164 | ≥16 | ≥16 | ≥64 | ≥64 | ≥64 | ≥64 | ≥4 | ≥8 | ≥128 | ≥64 | ≤0.5 | ≤0.5 | ≤20 | ≥16 | ≥16 |
| 68 | xz182 | ≥16 | ≥16 | ≥64 | ≥64 | ≥64 | ≤2 | ≥4 | ≥8 | ≥128 | ≥64 | ≤0.5 | 4 | ≥320 | ≤1 | ≤1 |
| 69 | xz058 | ≥16 | ≥16 | ≥64 | ≥32 | ≥64 | ≤2 | ≥4 | ≥8 | ≥128 | ≥64 | ≤0.5 | ≤0.5 | ≤20 | ≤1 | ≤1 |
| 70 | xz151 | ≥16 | ≥16 | ≥64 | ≥32 | ≥64 | ≥64 | ≥4 | ≥8 | ≥128 | ≥64 | ≥16 | ≤0.5 | ≥320 | NA | ≥16 |
| 71 | xz153 | ≥16 | ≥16 | ≥64 | ≥64 | ≥64 | NA | ≥4 | ≥8 | ≥128 | ≥64 | ≤0.5 | ≤0.5 | ≥320 | ≤1 | 8 |
| 72 | xz113 | ≥16 | ≥16 | ≥64 | ≥64 | ≥64 | ≤2 | ≥4 | ≥8 | ≥128 | ≥64 | ≤0.5 | ≤0.5 | ≤20 | ≤1 | ≤1 |
| 73 | xz056 | ≥16 | ≥16 | ≥64 | ≥32 | ≥64 | ≤2 | ≤0.25 | ≤0.25 | ≥128 | ≥64 | ≤0.5 | ≤0.5 | ≤20 | ≤1 | ≤1 |
| 74 | xz055 | ≥16 | ≥16 | ≥64 | ≥32 | ≥64 | ≤2 | ≤0.25 | ≤0.25 | ≥128 | ≥64 | ≤0.5 | ≤0.5 | ≤20 | ≤1 | ≤1 |
| 75 | xz122 | ≥16 | ≥16 | ≥64 | ≥64 | ≥64 | ≤2 | ≤0.25 | ≤0.25 | ≥128 | ≥64 | ≤0.5 | ≤0.5 | ≤20 | ≤1 | ≤1 |
| 76 | xz100 | 8 | ≥16 | ≥64 | ≥64 | ≥64 | NA | 1 | 1 | ≥128 | ≥64 | ≤0.5 | ≤0.5 | ≥320 | ≤1 | 8 |
| 77 | xz088 | ≥16 | ≥16 | ≥64 | ≥32 | ≥64 | ≤2 | ≤0.25 | ≤0.12 | ≥128 | ≥64 | ≤0.5 | ≤0.5 | ≤20 | ≤1 | ≤1 |
| 78 | xz073 | ≥16 | ≥16 | ≥64 | ≥32 | ≥64 | ≤2 | ≤0.25 | ≤0.12 | ≥128 | ≥64 | ≤0.5 | ≤0.5 | ≤20 | ≤1 | ≤1 |
| 79 | xz060 | ≥16 | ≥16 | ≥64 | ≥32 | ≥64 | ≤2 | ≤0.25 | ≤0.25 | ≥128 | ≥64 | ≤0.5 | ≤0.5 | ≤20 | ≤1 | ≤1 |
| 80 | xz134 | ≥16 | ≥16 | ≥64 | ≥32 | 16 | ≤2 | ≤0.25 | ≤0.25 | 64 | ≥64 | ≤0.5 | ≤0.5 | ≤20 | ≤1 | ≤1 |
| 81 | xz091 | NA | NA | NA | NA | NA | NA | NA | NA | NA | NA | NA | NA | NA | ≤1 | NA |
| 82 | xz052 | 8 | ≥16 | ≥64 | ≥32 | ≥64 | ≤2 | ≤0.25 | 1 | ≥128 | ≥64 | ≤0.5 | ≤0.5 | ≤20 | NA | NA |
| 83 | xz064 | ≥16 | ≥16 | ≥64 | ≥32 | ≥64 | ≤2 | ≥4 | 1 | ≥128 | ≥64 | ≤0.5 | ≤0.5 | ≥320 | ≤1 | 8 |
| 84 | xz130 | ≥16 | ≥16 | ≥64 | ≥64 | ≥64 | ≤2 | ≤0.25 | 1 | ≥128 | ≥64 | ≤0.5 | ≤0.5 | ≤20 | ≤1 | ≤1 |
| 85 | xz108 | ≥16 | ≥16 | ≥64 | ≥64 | ≥64 | ≤2 | ≤0.25 | 1 | ≥128 | ≥64 | ≤0.5 | ≤0.5 | ≤20 | ≤1 | ≤1 |
| 86 | xz051 | ≥16 | ≥16 | ≥64 | ≥32 | ≥64 | ≤2 | 0.5 | 1 | ≥128 | ≥64 | ≤0.5 | 2 | ≤20 | NA | NA |
| 87 | xz077 | 8 | ≥16 | ≥64 | ≥32 | 4 | ≤2 | ≥4 | ≥8 | ≥128 | ≥64 | ≤0.5 | ≤0.5 | 40 | ≥16 | 2 |
| 88 | xz068 | 8 | ≥16 | ≥64 | ≥32 | ≤1 | ≤2 | 1 | 1 | ≥128 | ≥64 | ≤0.5 | ≤0.5 | ≤20 | ≥16 | 8 |
| 89 | xz084 | ≥16 | ≥16 | ≥64 | ≥32 | ≥64 | 8 | ≥4 | ≥8 | ≥128 | ≥64 | ≤0.5 | ≤0.5 | ≥320 | ≤1 | ≥16 |
| 90 | xz102 | 8 | ≥16 | ≥64 | ≥64 | 16 | ≤2 | 0.5 | 1 | 64 | ≥64 | ≤0.5 | ≤0.5 | ≤20 | ≤1 | ≤1 |
| 91 | xz053 | ≥16 | ≥16 | ≥64 | ≥32 | ≤1 | ≤2 | ≤0.25 | ≤0.12 | ≥128 | ≥64 | ≤0.5 | 2 | ≤20 | NA | NA |
| 92 | xz099 | ≥16 | ≥16 | ≥64 | ≥64 | 32 | ≤2 | ≤0.25 | ≤0.25 | ≥128 | ≥32 | ≤0.5 | ≤0.5 | ≤20 | NA | ≤1 |
| 93 | xz087 | ≥16 | ≥16 | ≥64 | ≥32 | 16 | ≤2 | 0.5 | 1 | ≥128 | ≥64 | ≤0.5 | ≤0.5 | ≥320 | ≥16 | 4 |
| 94 | xz063 | ≥16 | ≥16 | ≥64 | ≥32 | ≥64 | ≤2 | ≥4 | 4 | ≥128 | ≥64 | ≤0.5 | ≤0.5 | ≥320 | ≤1 | ≤1 |
| 95 | xz062 | 8 | ≥16 | ≥64 | ≥32 | ≥64 | ≤2 | 2 | 4 | ≥128 | ≥64 | ≤0.5 | ≤0.5 | ≥320 | ≤1 | ≤1 |
| 96 | xz070 | ≥16 | ≥16 | ≥64 | ≥32 | ≥64 | ≤2 | ≥4 | NA | NA | ≥64 | ≤0.5 | ≤0.5 | ≤20 | ≥16 | NA |
| 97 | xz177 | 8 | ≥16 | ≥64 | ≥64 | ≤1 | ≤2 | ≥4 | ≥8 | 8 | ≥64 | ≤0.5 | ≤0.5 | ≤20 | ≤1 | ≤1 |
| 98 | xz105 | 8 | ≥16 | ≥64 | ≥64 | 16 | ≤2 | 0.5 | 1 | ≥128 | ≥64 | ≤0.5 | ≤0.5 | ≥320 | ≥16 | 2 |
